# Challenges of Longevity: Safety of Vaginal and Laparoscopic Urogynecological Procedures in Septuagenarians and Older Patients

**DOI:** 10.1155/2016/5184595

**Published:** 2016-12-13

**Authors:** R. Joukhadar, A. Wöckel, D. Herr, V. Paulus, J. Radosa, A. Hamza, E. Solomayer, S. Baum

**Affiliations:** ^1^Department of Obstetrics and Gynecology, University of Würzburg, Würzburg, Germany; ^2^Department of Obstetrics and Gynecology, University of Saarland, Homburg, Germany; ^3^Department of Obstetrics and Gynecology, University of Schleswig-Holstein, Campus Lübeck, Lübeck, Germany

## Abstract

*Introduction*. Pelvic organ prolapse (POP) and urinary incontinence (UI) have increasing prevalence in the elderly population. The aim of this study was to compare the comorbidities of these procedures between <70 y/o and ≥70 y/o patients.* Materials and Methods*. In our retrospective study over a period of 2.5 years, 407 patients had received an urogynecological procedure. All patients with POP were treated by reconstructive surgery. Complications were reported using the standardized classification of Clavien-Dindo (CD). The study can be assigned to stage 2b Exploration IDEAL (Idea, Development, Exploration, Assessment, Long-term study)-system of surgical innovation.* Results*. Operation time, blood loss, and intraoperative complications have not been more frequent in the elderly, whereas hospital stay was significantly longer in ≥70 y/o patients. Regarding postoperative complications, we noticed that ≥70 y/o patients had an almost threefold risk to develop mild early postoperative complications compared to younger patients (OR: 2.86; 95% CI: 1.76–4.66). On the contrary, major complications were not more frequent. No case of life-threatening complication or the need for blood transfusion was reported.* Conclusion*. After urogynecological procedures, septuagenarians and older patients are more likely to develop mild postoperative complications but not more intraoperative or severe postoperative complications compared to younger patients.

## 1. Introduction

Both UI and POP are pelvic floor dysfunctions frequently encountered in older women [1]. Recent data revealed high prevalence of both entities with a peak at the age of 70-71 years for UI and a progressively increasing age-specific annual risk for POP [2].

Taking demographic trends into account, it is clear that there is a global significant increase in longevity, which is also to be noticed in Germany [3, 4]. The greater life expectancy for women leads to a sex ratio increase with age. This trend underpins the inexorably expected growing need for treatment modalities in this population [5].

There seems to be a very negative impact of POP and urinary incontinence on women's quality of life, social behavior, and even their mental status [6, 7]. It is estimated in Germany that up to 50% of admissions to nursing homes take place for burdens related to urinary incontinence [8].

Although pessaries are not a causal treatment and are often associated with discomfort, their use is still frequent in the elderly [9, 10]. In many cases, however, surgical correction is the only way to restore anatomy and function. Yet, the elderly are often regarded as unfavorable clientele and are denied access to surgical intervention due to their higher age.

When surgery is performed, obliterative procedures remain more frequently applied [11, 12]. Yet, evidence suggests the equality and even superiority of reconstructive procedures [13, 14].

Although urogynecological surgery in the elderly seems a pressing public health issue, age-related perioperative comorbidity especially in reconstructive prolapse surgery is underreported. Our aim was to compare perioperative morbidity in septuagenarians and older patients undergoing surgery with that of younger ones in a retrospective single-center study and to estimate the safety of applying reconstructive procedures in the elderly.

## 2. Materials and Methods

### 2.1. Study Design

We conducted a single-center retrospective study at the University Hospital of Saarland in Homburg, Germany, which is a tertiary referral center for both gynecologic laparoscopy and urogynecology to compare the perioperative morbidities associated with urogynecological procedures regarding the age of patients. Data were collected by reviewing the electronic patient's charts.

All patients who had undergone an operative procedure for treatment of POP or UI between July 2012 and December 2014 were continuously enrolled in this study. In this period of 2.5 years, overall 407 patients were surgically treated. We chose an age cutoff of 70 years and regarded patients ≤70 years as younger, while those aged ≥70 years were regarded as older patients.

Our presented study can be assigned to stage 2b Exploration IDEAL (Idea, Development, Exploration, Assessment, Long-term study)-system of surgical innovation. This stage focuses on adverse effects and potential benefits.

### 2.2. Collected Data

In addition to demographic data, we collected detailed obstetric and surgical history. In order to assess the effect of comorbidities, we chose the American Society of Anesthesiologists' (ASA) risk classification system as an index for the general condition of elderly patients.

We further collected intraoperative data regarding duration of surgery and intraoperative complications as well as postoperative data including hemoglobin (Hb) decline, hospital stay, and the occurrence and type of postoperative complications. We defined Hb decline as the difference between preoperative Hb and the lowest Hb measured during postoperative hospital stay. Details to the evaluation of postoperative complications are presented in Section 2.4.

As for laparoscopies, we regarded the conversion to laparotomy for any reason an intraoperative complication and documented it as such.

### 2.3. Applied Procedures and Materials

Cases of UI were surgically treated using retropubic and transobturator slings as well as laparoscopic Burch colposuspension. Although technically more challenging, all patients suffering from POP were offered reconstructive procedures. Applied POP surgery can be further subdivided into native tissue repair and mesh-assisted repair.

There was no need to perform obliterative vaginal surgery or abdominal procedures on the patients enrolled in this study, as they could all be treated with reconstructive vaginal and laparoscopic procedures, which indicates the high standard of treatment.

Vaginal native tissue repair offered included anterior and/or posterior colporrhaphy, McCall culdoplasty procedure, transvaginal sacrospinous fixation, or a combination of these procedures as indicated. Mesh-assisted repair mainly included laparoscopic sacropexy in addition to anterior, posterior, or total vaginal mesh in some selected cases.

Applied mesh material consisted of macroporous type-1 meshes made of polypropylene in most cases and polyvinylidenfluorid (PVDF) in some cases.

### 2.4. Presentation of Postoperative Complications

Postoperative complications were subdivided into early and intermediate depending on the time of occurrence. Those taking place from leaving the operation room (OR) to 72 h after discharge were regarded as early complications and those occurring from 72 h to 30 days after discharge were regarded as intermediate ones. The minimum follow-up time was 30 days after discharge.

We applied the standardized CD classification to record the postoperative complications in this study. This classification provides uniform definitions for the existence and severity of a surgical complication.

Since complications grades CD-I and CD-II represent those managed nonoperatively and only differ in the type of management required, we regarded the sum of both as mild complications. On the other hand, we regarded the sum of complications grade CD-IIIa (complications that required operative management under local anesthesia) and grade CD-IIIb (complications that required operative management under general anesthesia) as severe complications.

We compared the occurrences of postoperative complications for both the early and intermediate time intervals. In each of these intervals, the comparison was undertaken regarding each complication grade on its own (CD-I, CD-II, CD-IIIa, and CD-IIIb), as well as regarding the sum of mild (CD-I + CD-II) and that of severe (CD-IIIa + CD-IIIb) complications.

### 2.5. Statistics

According to the intent-to-treat principle, all patients with evaluable data were analyzed using descriptive statistics. Missing data were not imputed. The null hypothesis of no difference between those younger than 70 years and septuagenarians or older patients receiving urogynecological procedure was exploratively tested against its alternative of any difference.

Categorical variables were tested using chi-square test and Fisher's exact test. Continuous variables were tested using either Student's *t*-test under the assumption of equal variances or Mann–Whitney *U* test. *p* values were adjusted on the basis of multiple testing corrections via false discovery rate (FDR) using R© Version 3.2.0. Statistical significance was defined as adjusted *p* value of ≤0.05. The statistical analyses were performed using the program IBM SAS (Version 2.2.; SAS Inc., Cary, NC, USA; http://www.sas.com/). Details are described in Tables 1 [Table tab2] [Table tab3] [Table tab4] [Table tab5]–6.

Additionally, following predictors were analyzed by logistic regression to assess their impact on postoperative complications: affiliation to the group of ≥70 y/o patients, obesity (Body Mass Index (BMI) > 30), multiparity (≥3 births), and ASA score III or IV. All predictors staying significant (*p* < 0.05) after adjusting for multiple testing have been additionally analyzed in a multivariate model.

## 3. Results

### 3.1. Patient's Characteristics

Out of 407 patients who had undergone a surgical treatment for POP, urinary incontinence, or both, 278 (68.3%) were younger than 70 y/o, whereas 129 (31.7%) were aged 70 years or older.

The preoperative general health condition of septuagenarians and older patients was significantly worse compared with younger patients. Older patients more frequently were classified ASA-III (37.5% versus 11.5%; *p* < 0.001) (Table 1).

There were no significant differences regarding BMI. Differences regarding obstetrical history are presented in detail in Table 1.

### 3.2. Intraoperative Data

No significant differences could be found between both age groups regarding duration of surgery or the occurrence of intraoperative complications (Table 2).

Overall intraoperative complications occurred in 2.5% of the younger patients and in 2.3% of the septuagenarians and older patients. Most of these complications seemed to be occurring sporadically without any tendency for repetition except for the bladder injury which was the most common intraoperative complication in both groups (Table 2).

### 3.3. Postoperative Data

Regarding Hb decline, there was no significant difference between both age groups, whereas hospital stay was significantly longer in septuagenarians and older patients (Table 3).

### 3.4. Postoperative Complications

The postoperative complications were recorded using the CD classification and categorized into CD-I, CD-II, CD-IIIa, and CD-IIIb. No complication severity in this study reached higher than stage CD-IIIb. A detailed presentation of the postoperative complications can be found in Table 4.

We found that ≥70 y/o patients suffered significantly more frequently from mild postoperative complications, which was mainly attributed to the occurrence of grade CD-II. No significant differences, however, could be found concerning severe complications.

**Figure 1:**
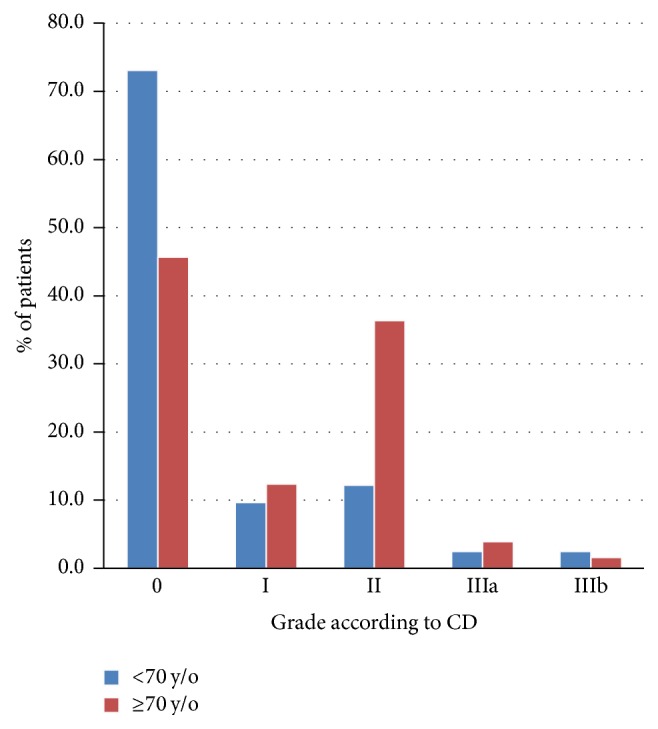
Early postoperative complications.

The incidence of early complications grade CD-II in ≥70 y/o patients was higher compared with younger patients (36.4% versus 12.2%; *p* < 0.001). Further significant differences were found regarding the sum of early mild complications (48.8% versus 21.9%; *p* < 0.001) (Table 5) (Figure 1).

**Figure 2:**
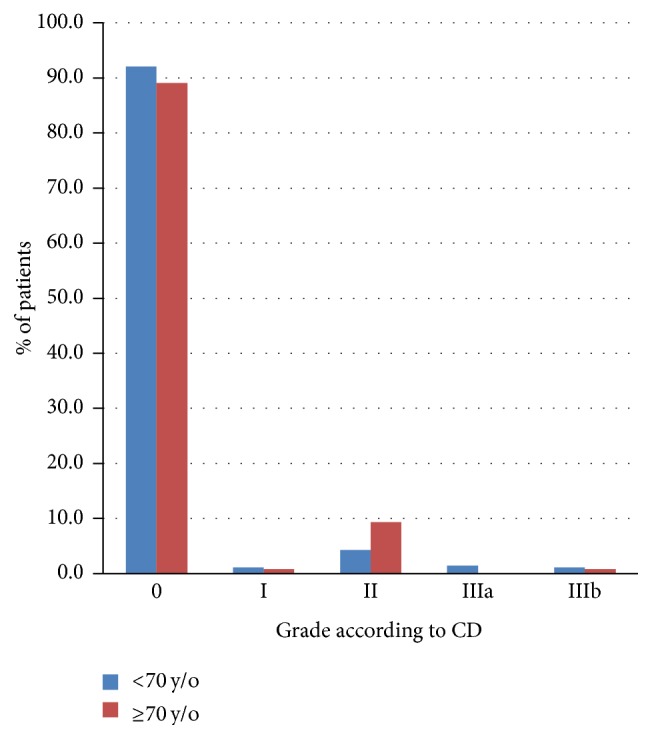
Late postoperative complications.

On the contrary, we found that neither mild nor severe intermediate postoperative complications were more frequent in septuagenarians and older patients (Table 5) (Figure 2).

Four parameters have further been analyzed by a logistic regression to assess their impact on postoperative complications. Of those four, the following three showed significant results: affiliation to the group of ≥70 y/o patients, multiparity (≥3 births), and having an ASA score III or IV (*p* < 0.05). With regard to the resulting odds ratio, the group of ≥70 y/o patients had an almost threefold risk to develop early postoperative complications as compared to the group of younger patients (OR: 2.95; 95% CI: 1.89–4.61). However, the influence of multiparity was slightly less (OR: 1.75; 95% CI: 1.11–2.77) (Table 6).

Further differentiating the early complications into the two subgroups, mild and severe early postoperative complications revealed the fact that both affiliation to the elderly group (OR: 2.86; 95% CI: 1.76–4.66) and multiparity (OR: 1.77; 95% CI: 1.08–2.88) were significant predictors for the mild early postoperative complications.

None of the predictors showed a significant correlation to the occurrence of intraoperative complications, mild intermediate postoperative complications, or any severe postoperative complication (Table 6).

## 4. Discussion

Demographic data from Germany show that the proportion of people ≥65 y/o will rise from 21% in 2015 to an estimated 33% in 2060. Along with rising life expectancy, these trends are expected to increase the future need for urogynecological surgery [4].

Our contemporary data analysis shows that UI and POP in the elderly patients aged ≥70 years can be safely managed surgically and that, even in cases of total prolapse, reconstructive procedures can be applied with good outcome. Our choice of the age cutoff is based on the definition of geriatric patients in Germany, which is acknowledged to be a patient aged ≥70 y/o in addition to suffering from defined health burdens [15].

The percentage of ≥70 y/o patients in our study was impressively high compared with other studies with the same age cutoff (31.7% versus 21%, 18.8%, and 20.6%, resp.), which further underpins the study results [16–18].

Septuagenarians and older patients suffered significantly more often from a reduced general condition than the younger ones. Up to 38% of the elderly in our study had an ASA score of III-IV, yet they were offered the same surgical options as younger patients including reconstructive POP procedures.

We did not find significant differences concerning duration of surgery between both age groups, which was in consistence with data from previous studies [19, 20]. The occurrence of intraoperative complications also did not significantly differ between the age groups. This also seemed to agree with other studies [17, 19, 20].

The intraoperative complications in our study seemed to be sporadic occurring only once except for bladder injury which occurred in 6 patients of whom all had received a laparoscopic sacropexy. The incidence of bladder injury during laparoscopic sacropexy in our study was 6/104 (5.7%), which lays within the upper accepted range when compared with other studies [21, 22]. Amongst overall low incidence of complications in our study, this may be explained by the very low rate of conversion to laparotomy for technical difficulty in our study in comparison with reported rates in other studies (0% versus 1.9–4.6%) [21–23].

In order to evaluate surgical blood loss during surgery, we chose Hb decline rather than estimated blood loss for comparison, since it is less observer-dependent. Our data show low Hb decline in both age groups and no difference between them.

Hospital stay was significantly longer in ≥70 y/o patients than in the younger ones, which was in consistence with other studies [16, 18, 19]. Yet, a possible confounder in discharging elderly patients is one of logistic nature, like waiting for discharge solutions or assistance from social workers [24].

Age-dependent occurrence of postoperative complications after urogynecological procedures is controversially discussed in literature. Although fairly consistent in that a comparable anatomical outcome can be achieved, studies seem controversial with regard to postoperative complications. Whereas some showed no differences [16, 17, 25, 26], others had proven that elderly patients suffered significantly from more postoperative complications than the younger ones [18–20, 27].

In an effort to clarify this issue, we applied a very strict protocol for recording complications in which we defined a complication as any deviation whatsoever from the ideal perioperative course without judging the causality. Classifying complications according to CD is acknowledged in urogynecology and even recommended by medical societies [28–32].

The most prevalent complications grade CD-I were higher need for analgesics and prolonged urinary catheterization due to temporary urinary retention. Both occurred mainly as early complication. Since registration of higher need for analgesics requires a standardized postoperative pain management, it was not reported as a complication in most studies [18–20].

Regarding complications grade CD-II, the most prevalent ones were requirement of antibiotics and requirement of antihypertensives. The requirement of antibiotics in our study resulted in most cases from urinary tract infections (UTIs) which were detected postoperatively in 10% of ≥70 y/o patients and in 6.5% of the younger ones. This high prevalence of postoperative UTIs in urogynecological patients is in consistence with data from other studies [33].

Nevertheless, it has to be mentioned that we had performed a urinalysis in each operated patient on the first postoperative day during the removing of the indwelling catheter. Thus, the detection rate was very accurate even in asymptomatic patients. In their study, Sze at al. came to similar results [17].

The requirement of antihypertensives, which made up for the most obvious difference between both age groups, was solely underreported in most studies [17–20]. In a further evaluation of our clientele, we found that all the ≥70 y/o patients who had required antihypertensives postoperatively were known to suffer from hypertension preoperatively. These patients were already taking antihypertensive medication and experienced a rising need postoperatively, so that either the dosage of the patient's home medication had to be increased or an additional pharmacological substance is added.

Most of the mild complications in the elderly seem to be attributed to the preoperative comorbidity and reduced patient's mobility.

Comparing previously published data with our results is hampered by the lack of uniform methodology. Just to mention the age cutoff set for comparison, the definition of complications, and their classification, the follow-up time and the vast differences concerning the applied surgical procedures are only some differences that cannot be overcome.

The strengths of this study include the high percentage of ≥70 y/o patients compared with other studies, which further reaffirms the study results [16–18].

Another strength is the high ratio of reconstructive surgery of POP, which is technically more challenging but has anatomical and functional advantages towards obliterative procedures, which are more frequently applied in the elderly [11–14]. Thus, the data from our study encourage offering reconstructive procedures to elderly patients.

A further strength is that the study concerns the assignment to stage 2b Exploration IDEAL-system of surgical innovation. According to the definitions of the stages that were further explained on the website of the “IDEAL collaboration,” this stage involves data from studies with an output concerning measurement and comparison and focuses on adverse effects and potential benefits. Regarding the number of patients, it is stated that this stage has to involve many rather than few, and the number of surgeons is defined as many, too. In our study, we had involved more than 400 patients who were treated surgically by 4 surgeons.

The type of patients in the studies assigned to this stage 2b should be a mixed type with broadening of the indication. Our study seems to fulfill this requirement too, as the complete spectrum of accepted and standardized vaginal and laparoscopic reconstructive surgery was applied in our study. Our innovation concerned the application in a highly aged group of patients who are routinely denied access to these procedures because of lack of experience in the outcome in this age group [34, 35].

Concerning follow-up, stage 2b Exploration only requires short-term or patient-reported outcomes in opposition to stage 3 Assessment which requires middle-term and stage 4 which requires long-term outcomes. Although our study presents middle-term outcomes, it cannot be assigned to stage 3 Assessment, since either randomized clinical trials or multicenter data are required for this stage, which our data do not fulfil [34, 35].

Lastly, the study had continuously enrolled all performed procedures in this tertiary center without any exclusion criteria and the complications were recorded following a very strict and standardized protocol, leaving very little room for observer's interpretation.

But our study also had several limitations. The character of a monocentric case-control study implements a bias in the retrospective nature of data acquisition from possibly inhomogeneous documentation.

Another limitation is that patients were not randomized to the different procedures. It has rather to be said that the selection of the procedure for each patient was based on many criteria including age, which, in addition to the extent of the disease, patient's surgical history, and weight, was an important factor in decision making.

Lastly, there is a limitation regarding follow-up. All patients in whom an alloplastic material was applied during surgery were offered a follow-up at 3–6 months after discharge. This group of patients comprised 74.7% of whom almost 95% participated in follow-up. The remaining 5% were inquired by telephone. As for patients who had undergone native tissue repair, we assume that they would have sought treatment in our center in case of postoperative complications, since the hospital was the only regional tertiary center for urogynecology. Yet we can only be sure that the vast majority but not all possible complications are known to us.

In order to gain more valuable information about applying urogynecological surgery in the very aged, efforts should be undertaken to perform multicenter studies or randomized controlled studies to fulfil the requirements for assignment to stage 3 Assessment of IDEAL-system of surgical innovation. One further necessity seems to be constructing registries for further structured long-term follow-up and subgroup analysis according to IDEAL stage 5.

## 5. Conclusion

Even though the general health condition was significantly worse and the extent of prolapse was significantly higher in septuagenarians and older compared with younger patients, they were offered the same therapeutic options and treated using reconstructive POP surgery. Neither operation time nor blood loss or intraoperative complications were more frequent in ≥70 y/o patients, whereas hospital stay was significantly longer.

Regarding postoperative complications, we noticed that minor complications had occurred more frequently in ≥70 y/o patients who had an almost threefold risk to develop mild early postoperative complications compared with younger patients (OR: 2.86; 95% CI: 1.76–4.66). On the contrary, major complications were not more frequent. No case of life-threatening complication or the need for intensive care or blood transfusion was reported.

We advise that elderly patients with the need for urogynecological procedures should be offered all surgical options and counseled about a higher risk to develop minor but not major complications.

We advise to perform multicenter studies and to build up registries for further structured long-term follow-up and subgroup analysis.

## Figures and Tables

**Table 1:** Patient's characteristics.

Age group (y/o)	<70		≥70		
Parameter		[*n*]		[*n*]	*p* value
Age (y/o)	55.60 ± 8.94	[278]	75.41 ± 4.05	[129]	
BMI (kg/m^2^)	27.20 ± 4.72	[278]	26.97 ± 4.05	[129]	0.658^t^
ASA score		[278]		[128]	
I	31 (11.2%)		1 (0.8%)		0.001^c^
II	215 (77.3%)		78 (60.9%)		0.003^c^
III	32 (11.5%)		48 (37.5%)		<0.001^c^
IV	0 (0.0%)		1 (0.8%)		0.401^f^
Birth	2 [1-2]	[277]	2 [2-3]	[125]	0.032^m^
Vaginal delivery	2 [1-2]	[277]	2 [2-3]	[125]	0.005^m^
C-section	0 [0-0]	[277]	0 [0-0]	[125]	0.006^m^
Vac. extraction	0 [0-0]	[277]	0 [0-0]	[125]	0.098^m^
BW ≥ 4000 g	0 [0-0]	[277]	0 [0-0]	[125]	0.628^m^
BW ≥ 4500 g	0 [0-0]	[277]	0 [0-0]	[125]	0.717^m^
Multiparity (≥3)	68 (24.5%)	[277]	46 (36.8%)	[125]	0.029^c^

Grade IV prolapse (Baden-Walker) for each compartment					
Anterior	30 (15.6)	[192]	34 (28.8)	[118]	0.033^c^
Middle	11 (5.7)	[192]	28 (23.7)	[118]	< 0.001^c^
Posterior	7 (3.6)	[192]	9 (7.6)	[118]	0.328^c^

Data are presented as average ± standard deviation or median and [IQR].

Statistical test: t = *t*-test; m = Mann–Whitney *U* test; c = chi-squared test; f = Fischer's exact test.

Vac. extraction: vacuum extraction; BW: birth weight.

**Table 2 tab2:** Intraoperative data.

Age group (y/o)	<70		≥70		
		[*n*]		[*n*]	*p* value
Duration of surgery (min)					
All procedures	91.06 ± 65.57	[278]	96.64 ± 64.35	[129]	0.643^t^
POP procedures	109.32 ± 59.75	[167]	101.24 ± 60.93	[110]	0.643^t^
UI procedures	39.84 ± 35.09	[86]	33.09 ± 21.05	[11]	0.634^t^
Combined procedures	140 [67.50–219.50]	[25]	60.50 [40.75–233.75]	[8]	0.643^m^

Intraoperative complications	7 (2.5%)	[278]	3 (2.3%)	[129]	1^f^
Bladder injury	4 (57.1%)	[7]	2 (66.7%)	[3]	
Rectal injury	1 (14.3%)	[7]	0 (0.0%)	[3]	
Uterine perforation	0 (0.0%)	[7]	1 (33.3%)	[3]	
Emphysema	1 (14.3%)	[7]	0 (0.0%)	[3]	
High ventilation pressure	1 (14.3%)	[7]	0 (0.0%)	[3]	

Data are presented as average ± standard deviation or median and [IQR].

Statistical test: t = *t*-test; m = Mann–Whitney *U* test; f = Fischer's exact test.

**Table 3 tab3:** Postoperative data.

Age group (y/o)	<70		≥70		
		[*n*]		[*n*]	*p* value
Hb decline (g/dL)	1.13 ± 0.77	[247]	1.24 ± 0.89	[125]	0.343^t^
Hospital stay (day)	5 [3.50–7.00]	[278]	6 [2.25–8.75]	[129]	<0.001^m^

Hb: hemoglobin. Data are presented as average ± standard deviation or median and [IQR].

statistical test: t = *t*-Test; m = Mann-Whitney-*U* test.

**Table 4 tab4:** Detailed presentation of the postoperative complications.

Grade of complication	Age < 70	Age ≥ 70
Early complications, classified according to CD		

I	(i) Higher need for analgesics: 14	(i) Higher need for analgesics: 3
	(ii) Prolonged urinary catheterization: 3	(ii) Prolonged urinary catheterization: 4
	(iii) Requiring iv. fluids: 2	(iii) Requiring iv. fluids: 1
	(iv) Requiring vaginal tamponade: 1	(iv) Requiring vaginal tamponade: 2
	(v) Prolonged hospital stay: 1	(v) Prolonged hospital stay: 2
	(vi) Requiring observation in ICU: 3	(vi) Transient paresthesia: 1
	(vii) Requiring drugs for temporary symptomatic treatment: 3	(vii) Requiring drugs for temporary symptomatic treatment: 3

II	(i) Requiring antibiotics: 21	(i) Requiring antibiotics: 21
	(ii) Requiring antihypertensives: 6	(ii) Requiring antihypertensives: 21
	(iii) Requiring other drugs for temporary symptomatic treatment: 10	(iii) Requiring other drugs for temporary symptomatic treatment: 8

IIIa	*Performed under local anesthesia*	*Performed under local anesthesia*
	(i) Loosening a tight TVT sling: 6	(i) Loosening a tight TVT sling: 3
	(ii) Revision of a vaginal hematoma: 1	(ii) Revision of a vaginal hematoma: 1
		(iii) Suture of a scar dehiscence: 1

IIIb	*Performed under general anesthesia*	*Performed under general anesthesia*
	(i) Revision of a colporrhaphy scar: 4	(i) Revision of a colporrhaphy scar: 1
	(ii) Loosening a colposuspension suture: 1	(ii) Revision of a vaginal suture: 1
	(iii) Loosening a sacropexy mesh: 1	
	(iv) Revision of a rectal suture: 1	

Intermediate complications, classified according to CD		

I	(i) Higher need for analgesics: 1	(i) Observation/minor scar dehiscence: 1
	(ii) Observation/minor scar dehiscence: 1	
	(iii) Observation/minor urinary retention: 1	

II	(i) Requiring antibiotics: 8	(i) Requiring antibiotics: 9
	(ii) Requiring drugs for temporary symptomatic treatment: 4	(ii) Requiring drugs for temporary symptomatic treatment: 3

IIIa	*Procedures performed under local anesthesia*	
	(i) Draining a labial boil: 1	
	(ii) Loosening a tight TVT sling: 3	

IIIb	*Performed under general anesthesia*	*Procedures performed under general anesthesia*
	(i) Revision of a hematoma: 1	(i) Revision of a hematoma: 1
	(ii) Laparoscopic ureterolysis: 1	
	(iii) Loosening a colposuspension suture: 1	

**Table 5 tab5:** Postoperative complications classified according to Clavien-Dindo.

	Minor complications			Major complications		
Grade	I	II	(I + II)	IIIa	IIIb	(IIIa + IIIb)
Early compl.						
Age < 70	27 (9.7%)	34 (12.2%)	61 (21.9%)	7 (2.5%)	7 (2.5%)	14 (5.0%)
Age ≥ 70	16 (12.4%)	47 (36.4%)	63 (48.8%)	5 (3.9%)	2 (1.6%)	7 (5.4%)
*p value*	*0.796* ^c^	*<0.001* ^c^	*<0.001* ^c^	*0.796* ^f^	*0.967* ^f^	*1* ^c^

Late compl.						
Age < 70	3 (1.1%)	12 (4.3%)	15 (5.4%)	4 (1.4%)	3 (1.1%)	7 (2.5%)
Age ≥ 70	1 (0.8%)	12 (9.3%)	13 (10.1%)	0 (0.0%)	1 (0.8%)	1 (0.8%)
*p value*	*1* ^f^	*0.272* ^c^	*0.281* ^c^	*0.749* ^f^	*1* ^f^	*0.796* ^f^

Early compl.: early complications from leaving the OR until 72 hrs after discharge from hospital; late compl.: late complications occurring from 72 hrs until 30 days after discharge from hospital.

Statistical test: c = chi-squared test; f = Fischer's exact test.

**Table 6:** Postoperative complications: logistic regression and multivariate analysis.

	*p* value	*p* value	OR	95% CI
	Univariate analysis	Multivariate analysis		
*Early postoperative complications*				
Age group^1^	<0.001	<0.001	2.953	1.893–4.607
BMI	0.543		1.014	0.970–1.060
Multiparity^2^	0.005	0.035	1.746	1.102–2.769
ASA score^3^	0.002	0.140	1.560	0.912–2.669

Mild early complications				
Age group^1^	<0.001	<0.001	2.862	1.757–4.661
BMI	0.388		1.021	0.974–1.070
Multiparity^2^	0.005	0.046	1.765	1.082–2.880
ASA score^3^	<0.001	0.064	1.748	1.004–3.043

Severe early complications				
Age group^1^	0.627		1.720	0.664–4.459
BMI	0.782		0.971	0.873–1.079
Multiparity^2^	0.627		1.631	0.630–4.224
ASA score^3^	0.935		0.949	0.267–3.374

*Intermediate postoperative complications*				
Age group^1^	0.666		1.417	0.700–2.868
BMI	0.674		1.025	0.953–1.103
Multiparity^2^	0.799		0.902	0.407–1.996
ASA score^3^	0.666		0.476	0.163–1.386

Mild intermediate complications				
Age group^1^	0.385		1.929	0.889–4.186
BMI	0.784		1.023	0.942–1.112
Multiparity^2^	0.086		0.923	0.377–2.260
ASA score^3^	0.784		0.634	0.214–1.882

Severe intermediate complications				
Age group^1^	0.997		0.318	0.039–2.615
BMI	0.997		1.033	0.891–1.198
Multiparity^2^	0.997		0.835	0.166–4.203
ASA score^3^	0.997		0.000	

OR: odds ratio; CI: Confidence Interval.

ASA score: for the logistic regression we summed up ASA I + II as well as ASA III + IV.

1 = Reference: age < 70 y/o; 2 = Reference: <3 births; 3 = Reference: ASA I + II.

*p* value was adjusted to “FDR.”

## Abbreviations

ASA: American Society of Anesthesiologists
BMI: Body Mass Index
CD: Clavien-Dindo
FDR: False discovery rate
Hb: Hemoglobin
IDEAL: Idea, Development, Exploration, Assessment, Long-term study
OR: Operation room
POP: Pelvic organ prolapse
UI: Urinary incontinence
UTI: Urinary tract infection.
